# Demonstration of In‐Memory Biosignal Analysis: Novel High‐Density and Low‐Power 3D Flash Memory Array for Arrhythmia Detection

**DOI:** 10.1002/advs.202308460

**Published:** 2024-05-06

**Authors:** Jangsaeng Kim, Jiseong Im, Wonjun Shin, Soochang Lee, Seongbin Oh, Dongseok Kwon, Gyuweon Jung, Woo Young Choi, Jong‐Ho Lee

**Affiliations:** ^1^ Department of Electrical and Computer Engineering and Inter‐university Semiconductor Research Center Seoul National University Seoul 08826 Republic of Korea; ^2^ Ministry of Science and ICT Sejong 30121 Republic of Korea

**Keywords:** 3D flash memory, computing‐in‐memory (CIM), electrocardiogram (ECG), neuromorphic, smart healthcare system

## Abstract

Smart healthcare systems integrated with advanced deep neural networks enable real‐time health monitoring, early disease detection, and personalized treatment. In this work, a novel 3D AND‐type flash memory array with a rounded double channel for computing‐in‐memory (CIM) architecture to overcome the limitations of conventional smart healthcare systems: the necessity of high area and energy efficiency while maintaining high classification accuracy is proposed. The fabricated array, characterized by low‐power operations and high scalability with double independent channels per floor, exhibits enhanced cell density and energy efficiency while effectively emulating the features of biological synapses. The CIM architecture leveraging the fabricated array achieves high classification accuracy (93.5%) for electrocardiogram signals, ensuring timely detection of potentially life‐threatening arrhythmias. Incorporated with a simplified spike‐timing‐dependent plasticity learning rule, the CIM architecture is suitable for robust, area‐ and energy‐efficient in‐memory arrhythmia detection systems. This work effectively addresses the challenges of conventional smart healthcare systems, paving the way for a more refined healthcare paradigm.

## Graphical abstract

This study introduces a novel 3D AND‐type flash memory for computing‐in‐memory architecture, enhancing the efficiency of smart healthcare systems. The fabricated high‐density and low‐power array effectively emulates biological synapses. The proposed architecture is suitable for robust, area‐ and energy‐efficient in‐memory arrhythmia detection systems, enabling timely detection of potentially life‐threatening arrhythmias. This research paves the way for more sophisticated healthcare solutions.

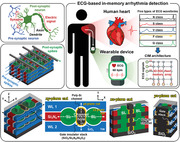

## 1 Introduction

Cardiovascular diseases (CVDs) constitute a significant portion of mortalities worldwide.^[^ ^1^, ^2^, ^3^, ^4^ ^]^ Timely identification is paramount in mitigating or managing the progression of CVDs effectively. Cardiac arrhythmias commonly serve as indicators for various CVDs. Since the arrhythmias are manifested in the electrical activities of the heart, electrocardiogram (ECG) signals prove invaluable in their detection. ECG signal has long been utilized as a reliable metric to monitor the functionality of the cardiovascular system.^[^ ^2^, ^5^, ^6^ ^]^ It provides insight into the intricate dynamics of the human heart due to its simplicity and noninvasive characteristics. This emphasizes the importance of consistent ECG monitoring for preemptive identification of potential CVDs.^[^ ^4^, ^7^, ^8^ ^]^ Nowadays, personal wearable electronic devices, which symbolize the combination of technology and medicine, provide real‐time continuous ECG monitoring in daily life.^[^ ^3^, ^7^ ^]^ While the ECG signals play a pivotal role in diagnosing arrhythmias, subtle differences between the ECG waveforms make it difficult to detect cardiac irregularities.^[^ ^9^, ^10^, ^11^, ^12^, ^13^ ^]^ However, advances in deep neural networks (DNNs) have enabled the efficient detection and classification of arrhythmias, a historically difficult task that requires meticulous analysis of each ECG waveform.^[^ ^3^, ^12^, ^13^, ^14^, ^15^, ^16^, ^17^, ^18^, ^19^, ^20^ ^]^

In recent years, DNNs have garnered significant attention for their unprecedented capabilities across diverse domains, notably in image classification, natural language processing, and healthcare.^[^ ^21^, ^22^, ^23^, ^24^ ^]^ However, the conventional von Neumann computing architecture, foundational to many of these systems, has limitations in terms of area occupancy and power consumption.^[^ ^25^, ^26^, ^27^, ^28^, ^29^, ^30^ ^]^ This is primarily attributed to the intense data communication demands between processing units and memory.^[^ ^28^, ^29^, ^30^ ^]^ In particular, the ECG classification processors for wearable devices encounter two challenges: the necessity of high area and energy efficiency while maintaining high classification accuracy.^[^ ^31^ ^]^ In this regard, conventional von Neumann computing architecture is unsuitable for processing enormous amounts of ECG signals in wearable devices, where miniaturization and low‐power operation are indispensable. Furthermore, necessitating data communications with external resources for ECG signal processing could impose additional hardware demands and communications networks.^[^ ^4^ ^]^ Therefore, robust, energy‐efficient, and high‐density computing architectures for wearable devices are essential to process and classify vast amounts of ECG signals in real‐time without external data communication.

**Figure 1:**
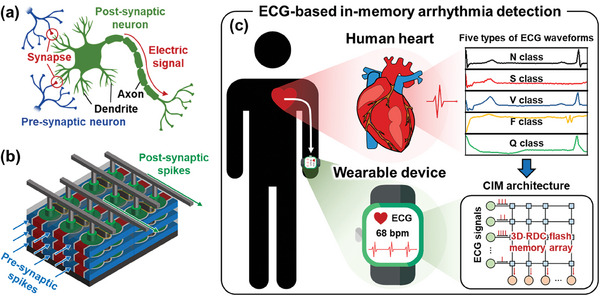
Proposed ECG‐based in‐memory arrhythmia detection system. a) Schematic illustration of the biological nervous system consisting of synapses and neurons. b) Schematic view of the proposed 3D AND‐type rounded dual channel (RDC) flash memory array for computing‐in‐memory (CIM) architecture. c) Schematic image of proposed ECG‐based in‐memory arrhythmia detection system using the 3D RDC flash memory array for smart healthcare systems. The ECG signals from the user's heart are continuously monitored through a wearable device. The compact CIM architecture integrated with the wearable device automatically detects arrhythmias by identifying abnormal ECG signals.

Neuromorphic computing architectures, inspired by biological nervous systems (**Figure** **1**a), have emerged as promising candidates to address the inherent constraints of the von Neumann computing architectures.^[^ ^32^, ^33^ ^]^ Many studies have been conducted to classify ECG signals utilizing this neuromorphic computing architecture.^[^ ^13^, ^14^, ^15^, ^16^, ^17^, ^18^ ^]^ While previously published studies achieved high classification accuracy using convolutional neural networks (CNNs),^[^ ^17^, ^18^, ^19^ ^]^ the area occupancy and power consumption are significant due to the large network size with multiple convolution layers. Furthermore, the reliance on the off‐chip training approach, which transfers pretrained weights to synaptic devices, not only introduces susceptibility to device‐to‐device variations but also presents constraints in addressing patient‐to‐patient variability. The patient‐to‐patient variability can result in significant disparities between the ECG signals of patients in the training dataset and those of actual users, potentially leading to a considerable decrease in classification accuracy.^[^ ^31^ ^]^

In neuromorphic computing architectures, the performance is significantly influenced by electronic synaptic devices mimicking biological synapses. A variety of emerging memory devices have been reported as synaptic devices, ranging from resistive random‐access memory (ReRAM)^[^ ^12^, ^34^, ^35^, ^36^, ^37^, ^38^, ^39^ ^]^ and phase‐change memory (PCM)^[^ ^40^, ^41^, ^42^ ^]^ to flash memory.^[^ ^43^, ^44^, ^45^, ^46^, ^47^, ^48^, ^49^, ^50^, ^51^ ^]^ Most of the reported synaptic devices are capable of performing efficient vector‐matrix multiplication (VMM) operations on an array. However, while ReRAM and PCM have the advantages of fabrication simplicity and high density, concerns regarding the reliability of the device remain. Notably, these encompass sneak path issues,^[^ ^52^, ^53^, ^54^, ^55^ ^]^ device‐to‐device variation,^[^ ^56^, ^57^ ^]^ and a constrained on/off ratio,^[^ ^58^, ^59^, ^60^ ^]^ which pose challenges to their robust and consistent performance. The reliability issues of the synaptic devices are fatal to smart healthcare systems since misdiagnosis of CVD is directly related to mortality.^[^ ^61^ ^]^ Accurate identifications of abnormal ECG signals are critical to saving lives. From this perspective, flash memory with high technological maturity is considered the most suitable candidate for smart healthcare systems. However, flash memory has limitations in terms of integration density compared to two‐terminal devices, such as ReRAM and PCM. To address this challenge, considerable research has been focused on 3D vertical stacked flash memory with high density.^[^ ^45^, ^46^, ^47^, ^48^, ^49^, ^50^, ^51^ ^]^

Two types of 3D vertical stacked flash memory have been reported: NAND‐type and AND‐type. While NAND‐type flash memory exhibits high density, it experiences extended latency due to the serial structure.^[^ ^44^, ^45^, ^46^ ^]^ Additionally, sequential floor‐by‐floor operations are required for neuromorphic systems.^[^ ^44^ ^]^ Contrasting with the NAND‐type flash memory, the AND‐type flash memory enables high‐speed operation thanks to the parallel current summation.^[^ ^46^, ^47^, ^48^, ^49^, ^50^ ^]^ In particular, unlike conventional planar structures,^[^ ^50^, ^51^ ^]^ a 3D AND‐type flash memory with a rounded channel enhances gate controllability.^[^ ^46^, ^47^ ^]^ Although they demonstrate multiple floors and stable memory operation characteristics, reducing the radius of the channel hole is constrained due to the presence of a plug‐shaped bit‐line (BL) and source‐line (SL) inside the channel hole. Consequently, a single device on each floor occupies a relatively large area. The other reported structures feature a gate electrode inside the plug surrounded by the channel.^[^ ^48^, ^49^ ^]^ These structures result in a concave channel, opposite to the gate‐all‐around (GAA) structure, and lead to a relatively low gate controllability.

In this work, we propose a novel 3D AND‐type flash memory array with a rounded double channel (RDC) for smart healthcare systems (Figure 1b). The proposed 3D RDC flash memory array exhibits high density with its capacity for easy multiple‐floor stacking. Two independent flash memory cells per floor for a single channel hole further improve cell density. A gate structure covering the convex channel enhances gate controllability. A simplified spike‐timing‐dependent plasticity (STDP) learning rule, which is simple and efficient to implement in hardware neural networks (HNNs) with a small size,^[^ ^62^, ^63^, ^64^ ^]^ is utilized for the ECG classification task. The simplified STDP learning rule and fully connected single‐layer HNN significantly reduce area occupancy and power consumption while maintaining high classification accuracy since no convolution layers exist. Additionally, the on‐chip training approach with the simplified STDP learning rule, which does not require weight transfer, can mitigate vulnerability to patient‐to‐patient variability and ensure consistent classification accuracy. Considering these perspectives, the 3D RDC flash memory array combined with the simplified STDP learning rule is suitable for robust, energy‐efficient, and high‐density computing‐in‐memory (CIM) architecture for arrhythmia detection (Figure 1c). The fabricated 3D RDC flash memory array is meticulously evaluated regarding synaptic memory characteristics and array operations for HNNs. The key fabrication process steps and structural modifications for the proposed flash memory are introduced. The 3D RDC flash memory array successfully classifies analogous ECG signals with high area and energy efficiency.

## 2 Results and Discussion

### 2.1 Flash Memory Cell Structure and Operations

**Figure 2:**
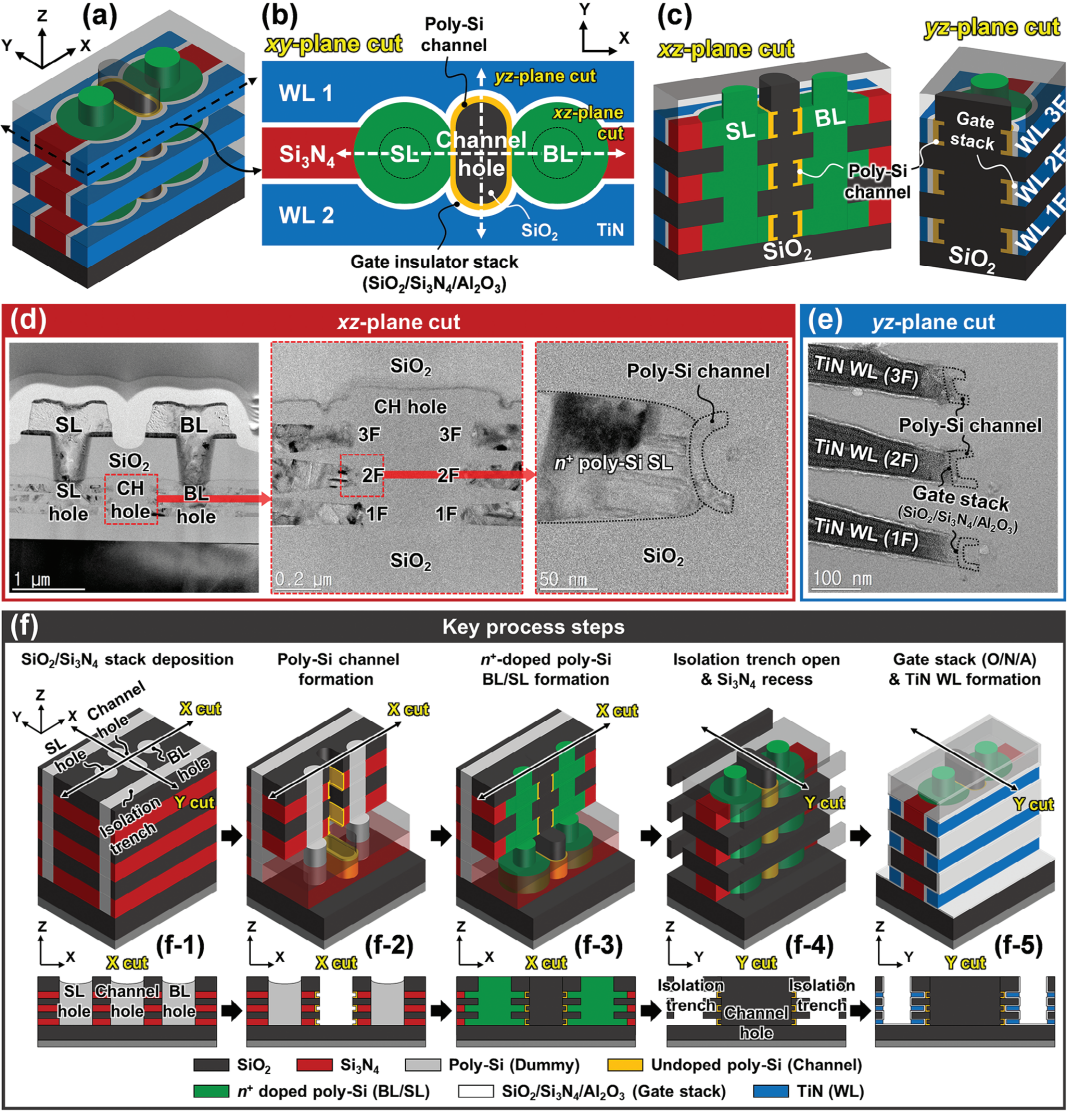
Proposed 3D RDC flash memory cell. a) Schematic view of the 3D RDC flash memory cells with three floors. b) Top and c) side cross‐sectional schematic views of the cells. TEM cross‐sectional images of the fabricated cells cut in the d) *xz*‐ and e) *yz*‐plane direction across the channel hole, respectively. Magnified TEM images verify the successful formation of the proposed cell structure. f) Schematic views of key process steps for fabricating proposed cells. The proposed cell demonstrates notable compatibility with the mature CMOS process technology thanks to the structural resemblance to the conventional V‐NAND.

**Figure** **2**a shows a schematic view of the 3D RDC flash memory cells with three floors. Figure 2b,c represent the top and side cross‐sectional schematic views of the cells, respectively. Around the ring‐shaped undoped poly‐Si channel, a high‐*κ* gate insulator stack consisting of SiO_2_/Si_3_N_4_/Al_2_O_3_ and metal gate electrodes (word lines; WLs) are formed. The proposed flash memory cell has a gate structure surrounding a convex thin poly‐Si channel, enhancing the gate controllability of the cell and reducing the program (PGM) and erase (ERS) voltage based on Fowler–Nordheim (FN) tunneling. A blocking oxide formed with Al_2_O_3_ reduces electron back tunneling through the oxide, improving memory efficiency.^[^ ^65^ ^]^ The separation of WLs on the same floor (WLs 1 and 2 in Figure 2b) by a Si_3_N_4_ region allows a single channel hole to operate individually as two flash memory cells for each floor. The cells on each floor operate completely independently since SiO_2_ layers separate each floor (Figure 2c). Unlike previous studies in which channels within a single vertical plug‐shaped channel hole were inevitably connected floor by floor during the fabrication process,^[^ ^46^, ^47^ ^]^ the channels separated by each floor minimize inter‐floor cell interference. On both sides of the channel along the *x*‐axis direction, the vertical plug‐shaped BLs and SLs are formed with *n* ^+^‐doped poly‐Si. Compared to the 2D array, the short cell distance in the 3D RDC flash memory array reduces the BL/SL resistance. Figure 2d,e shows transmission electron microscope (TEM) cross‐sectional images of the fabricated cells, which represent the *xz*‐ and *yz*‐plane across the channel hole, respectively. Magnified TEM images of the red dashed square region in Figure 2d are presented. The TEM images verify that the proposed cell structure is well‐formed.

**Figure 3:**
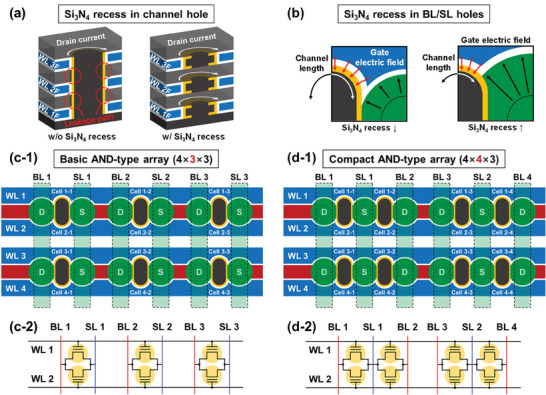
Structural modifications of 3D AND‐type RDC flash memory array. Formation of a) channel hole and b) BL/SL holes according to the Si_3_N_4_ recess process conditions in the fabrication process. Note that the presence of the Si_3_N_4_ recess process separates the channels within a single channel hole by each floor, while the absence of the process maintains the channels connected to floor by floor. The separated channels minimize inter‐floor cell interference. The Si_3_N_4_ recess process on the formation of the BL/SL holes affects the channel length. c‐1) Top schematic view and c‐2) circuit diagram of the 3D AND‐type RDC flash memory array with a basic structure. d‐1) Top schematic view and d‐2) circuit diagram of the 3D AND‐type RDC flash memory array with a compact structure. The compact structure achieves higher cell density by allowing two channel holes to share a single SL hole.

**Figure 4:**
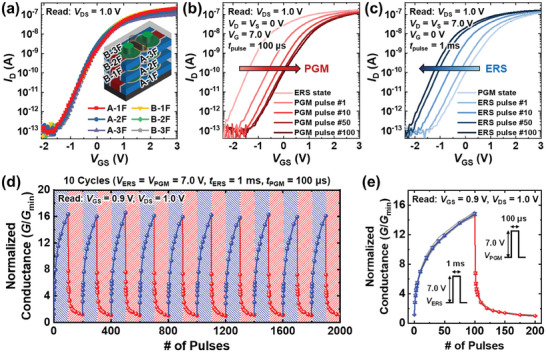
Electrical characteristics of 3D RDC flash memory cell. a) Transfer curves (*I*_D_–*V*_GS_) of the six fabricated 3D RDC flash memory cells in a single channel hole. Transfer curves with consecutive b) PGM and c) ERS pulses. The *V*_th_ of the device shifts depending on the number of applied pulses. d) Ten cycles of LTP and LTD characteristics of the fabricated cell with the number of applied pulses. The 200 pulses are applied during one cycle. The fabricated cell exhibits low cycle‐to‐cycle variation. e) Average values and standard deviations of both LTP and LTD characteristics of the fabricated cell with the number of applied pulses over ten cycles. Successive PGM and ERS pulses with identical amplitude were applied. The fabricated cell achieves multi‐level synaptic weights and shows excellent consistency.

**Figure 5:**
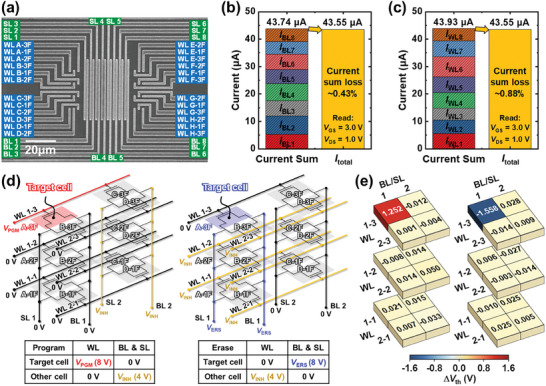
Electrical characteristics of 3D RDC flash memory array. a) Top SEM image of the fabricated 3D RDC flash memory array with a size of 8 × 8 × 3. Total array current (*I*_total_) and summed cell currents along the b) WLs and c) BLs in the 8 × 8 × 3 array, respectively. The difference between the *I*_total_ and the summed cell currents is less than 1%, verifying reliable and precise VMM operations. d) Schematic illustration and bias conditions for selective PGM/ERS operations. e) Changes in *V*_th_ of the target and inhibited cells. Only the target cell is programmed or erased while other cells are inhibited, verifying the random‐access capability.

Figure 2f illustrates the schematics and key steps of a fabrication process for the 3D RDC flash memory cells. The proposed cell exhibits significant compatibility with the mature CMOS process technology due to its comparable structure with conventional vertical NAND (V‐NAND). Following the sequential deposition of multiple SiO_2_/Si_3_N_4_ layers on the Si substrate, the active regions are patterned simultaneously using one mask. The patterned active regions are filled with dummy poly‐Si (Figure 2f‐1). This dummy poly‐Si later protects the remaining areas when partially processing the channel hole, BL/SL holes, and isolation trench in sequence. This process minimizes misalignment between the channel, BL/SL, and WLs, and reduces the device‐to‐device variation of flash memory cells that occurs during the fabrication process. After removing the dummy poly‐Si filled in the channel hole, the Si_3_N_4_ layers exposed on the inner surface of the channel hole are recessed by selective wet etching. An undoped poly‐Si channel is deposited, followed by anisotropic etching to separate the channels between floors. This process leaves only the channel deposited in the space where the Si_3_N_4_ layers are partially etched (Figure 2f‐2). SiO_2_ is deposited as a passivation layer of the separated channels. Subsequently, plug‐shaped BL/SL holes are formed through processes similar to those for channel hole formation. The deposition of an in situ *n* ^+^‐doped poly‐Si fills the BL/SL holes to form the BL/SL electrodes (Figure 2f‐3). The next step involves etching the dummy poly‐Si present in the isolation trench, followed by a recess of the Si_3_N_4_ layers through selective wet etching (Figure 2f‐4). Subsequently, the gate insulator stack (O/N/A) and the metal (TiN) as the gate material are deposited. An isotropic etching is performed to separate multiple TiN WLs for each floor (Figure 2f‐5). The back‐end‐of‐line (BEOL) process is executed as the final step. A more detailed fabrication process is described in the Experimental Section and Figures S1 and S2 (Supporting Information).

In the fabrication process, the selective wet etching of Si_3_N_4_ layers is a critical process that determines the electrical characteristics of the 3D RDC flash memory cells. Three Si_3_N_4_ recess processes are performed during the entire fabrication process: the channel hole, BL/SL holes, and isolation trench formation in sequence. When forming the isolation trench, the Si_3_N_4_ recess process is performed sufficiently so that the gate insulator stack and the WL completely cover the channel between the source and drain. This ensures high gate controllability for the channel. **Figure** **3**a,b represent the effect of the Si_3_N_4_ recess processes on the formation of the channel hole and BL/SL holes, respectively. When the Si_3_N_4_ recess is not performed while forming the channel hole, the channels within a single vertical plug‐shaped channel hole are connected floor by floor, as shown on the left of Figure 3a (refer to the process step in Figure 2f‐2). Therefore, leakage paths exist between cells on each floor, increasing inter‐floor cell interference. On the contrary, when the Si_3_N_4_ recess is performed, anisotropic etching of poly‐Si leaves only the channels in regions where the Si_3_N_4_ has been partially etched. As a result, the channels are separated by each floor, removing inter‐floor cell interference. The Si_3_N_4_ recess process on the formation of the BL/SL holes determines the length of the channel. As shown in Figure 3b, the cell on the left with a slightly etched Si_3_N_4_ has a relatively long channel length, while the cell on the right with a heavily etched Si_3_N_4_ has a short channel length. The Si_3_N_4_ recess process on the formation of the channel hole also contributes to the channel length. Note that the width of the channel can be adjusted by the thickness of the Si_3_N_4_ layers deposited in the early stage of the fabrication process.

In this work, the proposed 3D RDC flash memory cells have circular BL/SL holes and elliptical channel holes. The elongated channel hole in the *y*‐axis direction prevents BL and SL from being shorted with each other, which may occur when the Si_3_N_4_ layers are excessively wet etched in the BL/SL holes. As the length of the channel hole in the *y*‐axis direction becomes shorter, the wet etch margin of the Si_3_N_4_ layers for forming the BL/SL electrodes becomes smaller, and the risk of short circuits increases. In this work, long elliptical channel holes were formed to ensure sufficient wet etch margin of the Si_3_N_4_ layers, but the length of the channel hole in the *y*‐axis direction can be further minimized through fabrication process optimization.

The proposed 3D RDC flash memory cells are suitable for high‐density synaptic devices in neuromorphic systems since multiple floors can be easily stacked. The presence of the charge trap layer (Si_3_N_4_ layer of the gate insulator stack) facilitates the modulation of the synaptic device conductance through PGM and ERS operations. This enables the utilization of the synaptic device conductance as a weight in the neuromorphic systems. The proposed flash memory cell has the advantage of improving the cell density by separating a single channel hole into two independent cells per floor. Moreover, the cell density can be further enhanced by allowing two channel holes to share a single SL hole. Two possible AND‐type arrays (basic and compact structure) are shown in Figure 3c,d. Figure 3c illustrates a basic structure of the AND‐type array characterized by a single channel hole per SL hole. In Figure 3d, which depicts a compact structure, more cells are formed in the same area compared to the basic structure by sharing the same SL hole with neighboring cells. Note that the cell density of the proposed 3D RDC flash memory array can be further improved by reducing the gap between the BL/SL holes and the channel hole at improved technology nodes.

### 2.2 Electrical Characteristics of the Flash Memory Array

**Figure** **4**a shows the transfer curves (*I*_D_–*V*_GS_) of the six fabricated 3D RDC flash memory cells in a single vertical plug‐shaped channel hole. Moderate uniformity across the six cells is observed, characterized by a sub‐pA off current and on/off current ratio exceeding 10^5^. To assess the fundamental synaptic device characteristics of the fabricated cells, the transfer curves obtained by employing incremental step pulse programming/erasing (ISPP/ISPE) schemes are depicted in Figure S3 (Supporting Information). The ISPP measurement was conducted while increasing the programming voltage (*V*_G_ = *V*_PGM_ = 5.0–8.0 V, *V*_D_ = *V*_S_ = 0 V, *t* = 100 µs). Likewise, the ISPE measurement was conducted while increasing the erasing voltage (*V*_G_ = 0 V, *V*_D_ = *V*_S_ = *V*_ERS_ = 5.0–8.0 V, *t* = 10 ms). Both measurements were conducted under the condition of *V*_DS_ = 1.0 V. The fabricated cells are capable of low‐power PGM/ERS operations due to the PGM/ERS mechanism based on FN tunneling and the gate structure covering the convex thin poly‐Si channel. A reasonable program memory window is achieved.

In neuromorphic systems, the long‐term potentiation (LTP) and long‐term depression (LTD) characteristics are as important as the ISPP/ISPE characteristics, especially when performing on‐chip training. Figure 4b,c show the transfer curves with the successive PGM and ERS pulses under the condition of *V*_DS_ = 1.0 V. 100 identical PGM (*V*_GS_ = *V*_PGM_ = 7.0 V, *V*_D_ = *V*_S_ = 0 V, *t* = 100 µs) and ERS pulses (*V*_GS_ = 0 V, *V*_D_ = *V*_S_ = *V*_ERS_ = 7.0 V, *t* = 1 ms) are consecutively applied to the gate and source/drain of the cell, respectively. As the PGM and ERS pulses are applied, the trapping and de‐trapping of the charges in the charge trap layer cause the threshold voltage (*V*_th_) change of the fabricated cell. The ten cycles of LTP and LTD characteristics of the fabricated cell with the number of applied pulses are illustrated in Figure 4d. The fabricated cell shows nearly identical curves over ten cycles, verifying a consistent cycling performance. Low cycle‐to‐cycle variation (*σ*/*µ* < 0.02) contributes to the stability of the neuromorphic systems. Figure 4e represents the average values along with the standard deviations of both LTP and LTD characteristics of the fabricated cell with the number of applied pulses over ten cycles. Blue and red lines indicate average values and gray lines indicate individual LTP and LTD curves for ten cycles. Multi‐level synaptic weights and a *G*_max_/*G*_min_ ratio of greater than 15 are successfully obtained by applying identical PGM/ERS pulses. These characteristics enable the fabricated cells to emulate the features of biological synapses effectively and exhibit suitability for neuromorphic systems. The nonlinearity fitting parameters for LTP and LTD characteristics of the fabricated cell are investigated (Table S1, Supporting Information). The drain current (*I*_D_) fluctuation of the fabricated cell over time is depicted in Figure S4 (Supporting Information). The fabricated cell exhibits stable read operation characteristics for various *V*_GS_ conditions. The retention (>10^4^ s) and endurance (>10^4^ cycles) characteristics of the fabricated cell are further demonstrated (Figure S5, Supporting Information). The 200 PGM/ERS pulses are applied in one cycling test. The fabricated cell hardly deteriorates over 10^4^ PGM/ERS cycles.

The VMM operations and the selective PGM/ERS operations of the fabricated 3D RDC flash memory array are investigated. **Figure** **5**a shows the top scanning electron microscope (SEM) image of the fabricated 8 × 8 × 3 array. Figure 5b,c indicate the summed cell currents along the WLs and BLs in the fabricated 8 × 8 × 3 array, respectively. Each cell current is obtained by applying input signals only to each BL or WL. The sum of the cell currents separately obtained for each BL and WL is ≈43.74 and 43.93 µA, respectively. The total array current (*I*_total_) obtained by applying input signals to every BL and WL simultaneously is ≈43.55 µA. The difference between the total array current and the sum of each cell current is less than 1%. These results verify that the cell currents are accurately summed up along the BLs and WLs in the array, ensuring reliable and accurate VMM operations. High‐speed operation is capable due to the parallel weighted sum operation in the fabricated array.

To demonstrate the random‐access capability, the selective PGM/ERS operations were performed on the target cell. A schematic illustration and the corresponding bias conditions for the selective PGM/ERS operations are depicted in Figure 5d. In the selective PGM operation, *V*_PGM_ (8.0 V, 1 ms) is applied to the WL connected to the target cell, and 0 V is applied to the other WLs. While 0 V is applied to the BL and SL connected to the target cell, other cells in the array are inhibited by applying *V*_INH_ (4.0 V, 1 ms) to the other BLs and SLs. On the contrary, in the selective ERS operation, 0 V is applied to the WL connected to the target cell, and other cells in the array are inhibited by applying *V*_INH_ (4.0 V, 10 ms) to the other WLs. *V*_ERS_ (8.0 V, 10 ms) is applied to the BL and SL connected to the target cell, and 0 V is applied to the other BLs and SLs. The fabricated array has the advantage of exclusively using positive voltage for selective PGM/ERS operations. Figure 5e represents the changes in the *V*_th_ of the target and inhibited cells when the selective PGM/ERS operations are performed. All inhibited cells maintain their initial state while only the target cell is programmed or erased. The transfer curves with the selective PGM/ERS operations are shown in Figure S6 (Supporting Information). The difference in the current change between the target and inhibited cells is maintained within a 2% margin under the selective PGM/ERS conditions. These results verify the random‐access capability of the fabricated array. The transfer curves of the randomly selected 20 fabricated cells are presented in Figure S7 (Supporting Information).

### 2.3 In‐Memory ECG Classification

Smart healthcare systems necessitate the refined classification of the biosignals to facilitate accurate and real‐time diagnosis. In particular, precise and efficient classification of the ECG signals plays a pivotal role in arrhythmia detection. Given the significant resemblance between various ECG waveforms, an advanced methodology capable of discerning subtle differences is imperative. This study leverages HNN to classify the ECG waveforms effectively, offering an essential step toward a more intelligent healthcare landscape. The performance of the HNN employing the fabricated 3D RDC flash memory array is evaluated through the practical dataset: the MIT‐BIH Arrhythmia Dataset.^[^ ^66^, ^67^ ^]^ Based on different abnormal conditions in the human heart, the ECG waveforms can be categorized into five distinct types: N, S, V, F, and Q.^[^ ^19^ ^]^ The detailed annotations for these categories are outlined in Table S2 (Supporting Information).^[^ ^19^, ^68^ ^]^

**Figure 6:**
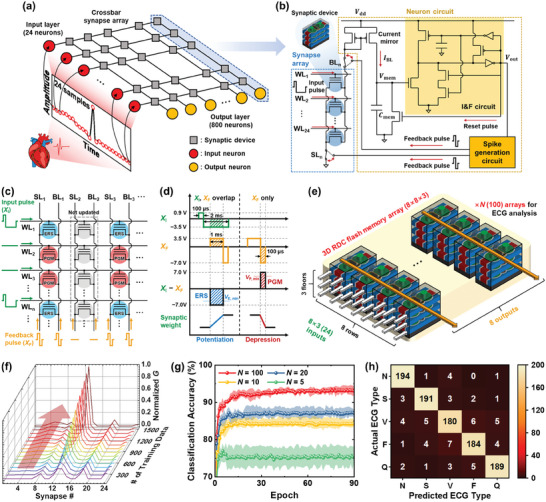
In‐memory ECG classification. a) Schematic illustration of the fully connected single‐layer HNN for the ECG classification. Five types of ECG waveforms are sampled and applied to the input layer. b) Proposed CIM architecture composed of 3D RDC flash synapse array and I&F neuron circuits. c,d) Pulse scheme for weight update following the simplified STDP learning rule. The overlapping interaction between the *X*_I_s and *X*_F_s facilitates the LTP/LTD operations, reflecting the simplified STDP learning rule. *V*_P, min,_ and *V*_E, min_ indicate the minimum voltage at which the synaptic device is programmed or erased, respectively. e) Unit 3D RDC flash synapse array (8 × 8 × 3) for the proposed HNN. Multiple unit synapse arrays constitute the entire HNN. f) Changes in the normalized conductance of the 24 synaptic devices as the training progresses. g) Classification accuracy for the ECG waveforms with the proposed CIM architecture by the training epoch. The classification accuracy increases as the training progresses. A higher average classification accuracy is obtained for the larger *N*. h) Confusion matrix for the ECG classification task. The proposed CIM architecture provides precise ECG waveform predictions, exhibiting minimal error.

**Figure** **6**a shows a schematic illustration of the fully connected single‐layer HNN for the ECG classification. The individual ECG waveforms extracted from continuous waveforms are sampled at 50 ms intervals. The electrical potential of these waves, consisting of 24 discrete time points, is applied to the input layer of the HNN. The HNN featured 24 input neurons corresponding to waveform sampling points and 800 output neurons representing different types of ECG waveforms, interconnected through a crossbar synapse array. Unlike previously published studies,^[^ ^13^, ^14^, ^15^, ^16^, ^17^, ^18^, ^19^ ^]^ a simplified STDP learning rule is employed without convolution layers rather than a backpropagation algorithm (Figure S8, Supporting Information). It is relatively simple to implement on a hardware basis and is efficient in terms of area and power consumption, making it suitable for the CIM architecture inside wearable devices. Moreover, given that the network training performs adequately with only positive weights, there is no need to employ inhibitory synaptic devices to represent negative weights, further enhancing area efficiency. Figure 6b illustrates a schematic circuit diagram of a proposed CIM architecture. The HNN consists of a synapse array utilizing a 3D RDC flash memory array and the integrate‐and‐fire (I&F) neuron circuits. The input pulses are applied to the WLs of the synapse array. The synaptic current flowing through the BLs (*I*_BL_) charges the membrane capacitor (*C*_mem_) of the neuron circuit. This facilitates a synaptic input integration operation. When the membrane potential (*V*_mem_) exceeds the specific threshold voltage, the I&F circuit generates an output pulse (*V*_out_). In the inference process, the output pulses are used to classify five types of ECG waveforms. The output pulses also act as reset pulses, which facilitate the discharge of the accumulated charge in the membrane capacitor, thereby reverting the membrane potential to its initial state. In the training process, the output pulses are transmitted to the spike generation circuit. The feedback pulses generated from the spike generation circuit are applied to the BLs/SLs of the synapse array. The feedback pulses cause conductance changes through PGM/ERS of the synaptic devices and implement weight update operation of the neural networks.

The PGM/ERS scheme of the synaptic devices for weight update following the simplified STDP learning rule is depicted in Figure 6c. While the input pulses (*X*_I_s) are applied to the WLs, the feedback pulses (*X*_F_s) are applied to the BLs/SLs of the synaptic array. When the BLs/SLs receive the *X*_F_s, the connected synaptic devices are programmed or erased depending on the presence of the *X*_I_s applied to the WLs: the presence of the *X*_I_s results in ERS, whereas the absence of the *X*_I_s results in PGM. On the contrary, the synaptic devices connected to the BLs/SLs without the *X*_F_s are not programmed or erased, regardless of the presence of the *X*_I_s. This PGM/ERS scheme is suitable for the simplified STDP learning rule and can be implemented through the overlapping interaction between the *X*_I_s and *X*_F_s. Figure 6d shows the pulse scheme for the simplified STDP learning rule. When the output neuron is fired through the application of the *X*_I_, the tail segment of the *X*_I_ overlaps the head segment of the *X*_F_. This is equivalent to applying a pulse of 7.0 V (*X*_I_−*X*_F_) to the BL/SL, resulting in the ERS of the synaptic device. This mimics the LTP operation of the synapse since the *X*_I_ is considered to have triggered the firing of the output neuron. On the contrary, when the output neuron is fired, even though the *X*_I_ is not applied, only the *X*_F_ is applied to the synaptic device. This corresponds to applying a pulse of 7.0 V (*X*_I_−*X*_F_) to the WL, leading to the PGM of the synaptic device. This mimics the LTD operation of the synapse since the *X*_I_ is considered to have not contributed to the firing of the output neuron. As previously verified in the selective PGM/ERS operations, the fabricated 3D RDC flash memory cells are hardly programmed or erased at voltages below 4.0 V (Figure 5d,e). This means that the synaptic device is not programmed or erased using the *X*_I_ alone (the amplitude of the *X*_I_ is below 4.0 V). Consequently, the absence of the *X*_F_s maintains the conductance of the synaptic devices, irrespective of the presence of the *X*_I_s (represented by the gray dotted line in Figure 6c). Figure 6e illustrates a unit synapse array of the single‐layer HNN employed for the ECG classification (Figure 6a). The unit synapse array is a fabricated 3D RDC flash memory array with a size of 8 × 8 × 3 (Figure 5a), which receives 24 inputs through the WLs and provides eight outputs through the SLs.

Multiple unit synapse arrays form the entire HNN, and the number of arrays required (*N*) varies depending on the network size. The ECG classification task is conducted for various *N*s and the performance depending on *N* is compared. Figure 6f represents the changes in the conductance (*G*) of the 24 synaptic devices connected to the single output neuron as the training progresses. The randomly distributed initial conductance is updated and represents a specific ECG waveform (here, class N as an example). The classification accuracy for the ECG waveforms with the proposed CIM architecture using the 3D RDC flash memory array is shown in Figure 6g. The ECG classification task was performed for various *N*s ranging from 5 to 100. To obtain the average classification accuracy, the performance evaluation was conducted on five distinct untrained networks for each *N*. The symbols indicate the average classification accuracy obtained through the five networks. This validates the uniformity of the results and mitigates any fluctuations or deviations that might be present in individual networks. The average classification accuracy of 93.5% is achieved for *N* = 100 (maximum classification accuracy of 94.6%). The confusion matrix between the actual and predicted types of ECG waveforms is also investigated (Figure 6h). The proposed CIM architecture is trained well and accurately predicts the actual types of the given ECG waveforms with minimal error despite the resemblance between the ECG waveforms. The t‐distributed stochastic neighbor embedding (t‐SNE) visualization, a dimension reduction and visualization technique for high‐dimensional data, of the samples from the MIT‐BIH dataset for the ECG classification is provided in Figure S9 (Supporting Information). Benchmarking results with prior works conducting ECG classification tasks using various nonvolatile memory are shown in Table S3 (Supporting Information). The proposed CIM architecture requires an energy consumption of 0.21 µJ for inferencing a single heartbeat signal (please see Note S1, Supporting Information, for the energy consumption). The results demonstrate that the CIM architecture employing the fabricated 3D RDC flash memory array and the simplified STDP learning rule is suitable for healthcare applications. It also exhibits a promising potential in diagnosing various diseases, including cardiac arrhythmia.

## 3 Conclusion

We have proposed a novel 3D AND‐type RDC flash memory array for a robust, area‐ and energy‐efficient in‐memory arrhythmia detection system. The proposed flash memory array exhibits high scalability with easily stackable multiple floors and two independent flash memory cells per floor for a single channel hole. The AND‐type array with a compact structure, sharing the same SL hole with neighboring cells, can further enhance cell density. The gate structure covering the convex thin poly‐Si channel improves gate controllability and facilitates low‐power PGM/ERS operations based on FN tunneling. Unlike previous studies, the channels separated by each floor minimize inter‐floor cell interference. The fabricated cells exhibit a sub‐pA off current and on/off current ratio exceeding 10^5^. Multi‐level synaptic weights are successfully obtained with low cycle‐to‐cycle variation (*σ*/*µ* < 0.02). The parallel weighted sum operation (with current sum error less than 1%) and the random‐access capability (with current variation in inhibited cells less than 2%) are investigated. The fabricated array mimics the features of biological synapses effectively. The high‐density and low‐power operation of the fabricated array ensures suitability for neuromorphic systems. A CIM architecture utilizing a 3D RDC flash memory array for in‐memory ECG classification is proposed and achieves high classification accuracy (93.5%), ensuring timely detection of potentially life‐threatening arrhythmias. A pulse scheme enabling simplified STDP learning rule is introduced.

The proposed CIM architecture has three significant advantages: 1) capability of accurate on‐device ECG signal analysis in real‐time without external data communications for off‐device analysis, 2) reduced area occupancy and power consumption due to the small size of the HNN and scalability of the 3D RDC flash memory array in the vertical direction, and 3) immunity to patient‐to‐patient variability attributed to the on‐chip training approach. Our work effectively addresses the limitations of the conventional ECG classification in smart healthcare systems and provides a pivotal advancement toward an enhanced healthcare paradigm. In smart healthcare systems, the advancement of neuromorphic computing architectures transcends mere ECG classification; it provides an essential instrument distinguishing normal cardiac activities from potentially life‐threatening arrhythmias.

## 4 Experimental Section

### Fabrication of 3D AND‐Type RDC Flash Memory Array

The 3D AND‐type RDC flash memory array was fabricated on a six‐inch Si wafer with ten masks. The key fabrication processes are as follows. First, a buried SiO_2_ layer was grown on the Si substrate. Next, multiple SiO_2_/Si_3_N_4_ layers (three layers) were sequentially deposited (Figure S1a, Supporting Information), and the active regions were simultaneously patterned with one mask (Figure S1b, Supporting Information). The dummy poly‐Si was deposited to fill the patterned active regions and etched for planarization (Figure S1c, Supporting Information). After removing the dummy poly‐Si filled in the channel hole, the Si_3_N_4_ layers exposed on the inner surface of the channel hole were recessed by selective wet etching (Figure S1d, Supporting Information). After an amorphous silicon (*a*‐Si) layer was deposited by low‐pressure chemical vapor deposition (LPCVD) process, an undoped poly‐Si channel was formed on the inner surface of the channel hole by re‐crystallizing the *a*‐Si layer at 600 °C for 24 h (Figure S1e, Supporting Information). The undoped poly‐Si channel (thickness of 12 nm) was separated by each floor through anisotropic etching (Figure S1f, Supporting Information), followed by SiO_2_ deposition to passivate each separated channel (Figure S1g, Supporting Information). After removing the dummy poly‐Si filled in the BL/SL holes, the Si_3_N_4_ layers exposed on the inner surface of the BL/SL holes were recessed by selective wet etching (Figure S1h, Supporting Information). The BL/SL electrodes were formed by depositing an in situ *n* ^+^‐doped poly‐Si in the exposed BL/SL holes and the space where the Si_3_N_4_ layers are partially recessed (Figure S1i, Supporting Information). Then the dummy poly‐Si filled in the isolation trench was removed (Figure S1j, Supporting Information). The Si_3_N_4_ layers exposed on the inner surface of the isolation trench were recessed by selective wet etching (Figure S1k, Supporting Information). The gate insulator stack was formed on the inner surface of the space where the Si_3_N_4_ layers were partially etched by sequentially depositing a layer of tunneling oxide (SiO_2_, 3 nm), nitride for the charge trap layer (Si_3_N_4_, 6 nm), and blocking oxide (Al_2_O_3_, 6 nm) (Figure S1l, Supporting Information). After depositing the metal (TiN) on the gate insulator stack as the gate material, multiple TiN metal gates separated by each floor were formed through isotropic etching (Figure S1m, Supporting Information). The isolation trench was filled with SiO_2_ for stack isolation, and then chemical mechanical polishing (CMP) was performed for planarization (Figure S1n, Supporting Information). Lastly, the BEOL process was executed. The whole fabrication process is represented in Figure S1 (Supporting Information).

During the fabrication process, the process for isolation trench separation (isolation cut) and WL contact (CT) pad formation was necessary. On both sides of the cell area, the area for the WL CT pads of each floor and isolation cut patterns exist (Figure S2a, Supporting Information). The isolation cut patterns separated the WL into two pieces for each floor, and were formed after channel formation (Figure S1f, Supporting Information) and before the BL/SL holes open process (Figure S1h, Supporting Information). The channel hole and isolation cut patterns were simultaneously filled with SiO_2_ (Figure S2b,c, Supporting Information). The schematic view of the 3D RDC flash memory cells with three WL CT pads is shown in Figure S2d (Supporting Information). Each WL CT pad provides a designated area for WL CT of each floor in the BEOL process. The fabrication process for WL CT pad formation is represented in Figure S2e (Supporting Information). This process was performed after BL/SL formation (Figure S1i, Supporting Information) and before WL formation (Figure S1m, Supporting Information).

Both the proposed 3D AND‐type RDC flash memory and the conventional V‐NAND flash memory can be easily multi‐floor structured, enabling the implementation of high‐density cells. While 3D RDC flash memory cells with three floors were fabricated in this work, 3D RDC flash memory cells with more floors could be fabricated by simply stacking more SiO_2_/Si_3_N_4_ layers. As the number of floors increases, the main challenge becomes the steep etching of vertical holes (maintaining a hole slope close to 90°) to minimize cell variation in the vertical direction. Through the optimization of the SiO_2_/Si_3_N_4_ layer thickness, it was feasible not only to mitigate these challenges but also to enhance both area and energy efficiency.

The proposed 3D AND‐type RDC flash memory cell was applicable to other nonvolatile memories besides flash memory. For example, 3D ferroelectric field‐effect‐transistors (FeFETs) could be fabricated by replacing the high‐*κ* gate insulator stack (SiO_2_/Si_3_N_4_/Al_2_O_3_) of the 3D RDC flash memory cell with a ferroelectric layer. Similarly, a resistance change layer or a phase change layer could also be applied to the proposed cell structure. The proposed cell structure had expandability to various emerging nonvolatile memories.

### Electrical Measurement and Network Structure

A probe station and a semiconductor parameter analyzer (B1500A) connected to the switching matrix were utilized to assess the synaptic memory characteristics of the fabricated 3D RDC flash memory array.

The network structure used in the ECG classification was the fully‐connected single‐layer HNN. The network had 24 input neurons and 8×*N* output neurons. The ECG signals utilized for the classification task were sourced from the MIT‐BIH Arrhythmia Database,^[^ ^66^ ^]^ courtesy of the Massachusetts Institute of Technology and Beth Israel Hospital via PhysioNet.^[^ ^67^ ^]^ Each ECG waveform, segmented from continuous signals, was sampled at intervals of 50 ms, yielding 24 discrete time points. The electrical potentials of the ECG waveforms were applied to the 24 input neurons corresponding to each sampling point. A simplified STDP learning rule, relatively simple to implement on a hardware basis compared to a backpropagation algorithm, was used for HNN training. The need for inhibitory synaptic devices to signify negative weights was eliminated. The homeostasis functionality was employed to improve the classification accuracy.^[^ ^64^, ^69^ ^]^

## Conflict of Interest

The authors declare no conflict of interest.

## Supporting information

Supporting Information

## Data Availability

The data that support the findings of this study are available in the supplementary material of this article.
