# Supplementary material for: Demonstration of In‐Memory Biosignal Analysis: Novel High‐Density and Low‐Power 3D Flash Memory Array for Arrhythmia Detection
Source: Adv Sci (Weinh). 2024 May 6;11(26):2308460. doi: 10.1002/advs.202308460 (PMC11234417; doi:10.1002/advs.202308460)
Supplement: Supplementary file 1 — Supporting Information [file ADVS-11-2308460-s001.pdf]

ADVANCED SCIENCE
Open Access

Supporting Information

for *Adv. Sci.*, DOI 10.1002/advs.202308460

Demonstration of In-Memory Biosignal Analysis: Novel High-Density and Low-Power 3D Flash Memory Array for Arrhythmia Detection

*Jangsaeng Kim, Jiseong Im, Wonjun Shin, Soochang Lee, Seongbin Oh, Dongseok Kwon, Gyuweon Jung, Woo Young Choi and Jong-Ho Lee**

Supporting Information

Demonstration of In-Memory Biosignal Analysis: Novel High-Density and Low-Power 3D Flash Memory Array for Arrhythmia Detection

Jangsaeng Kim[1], Jiseong Im[1], Wonjun Shin[1], Soochang Lee[1], Seongbin Oh[1], Dongseok Kwon[1], Gyuweon Jung[1], Woo Young Choi[1], and Jong-Ho Lee[1,2]*

[1] *Department of Electrical and Computer Engineering and Inter-university Semiconductor Research Center, Seoul National University, Seoul 08826, Republic of Korea*

[2] *Ministry of Science and ICT, Sejong 30121, Republic of Korea*

* Corresponding author. Tel.: +82-2-880-1727; Fax: +82 -2-882-4658.

E-mail: jhl@snu.ac.kr (J.-H. Lee)

Supplementary Figures

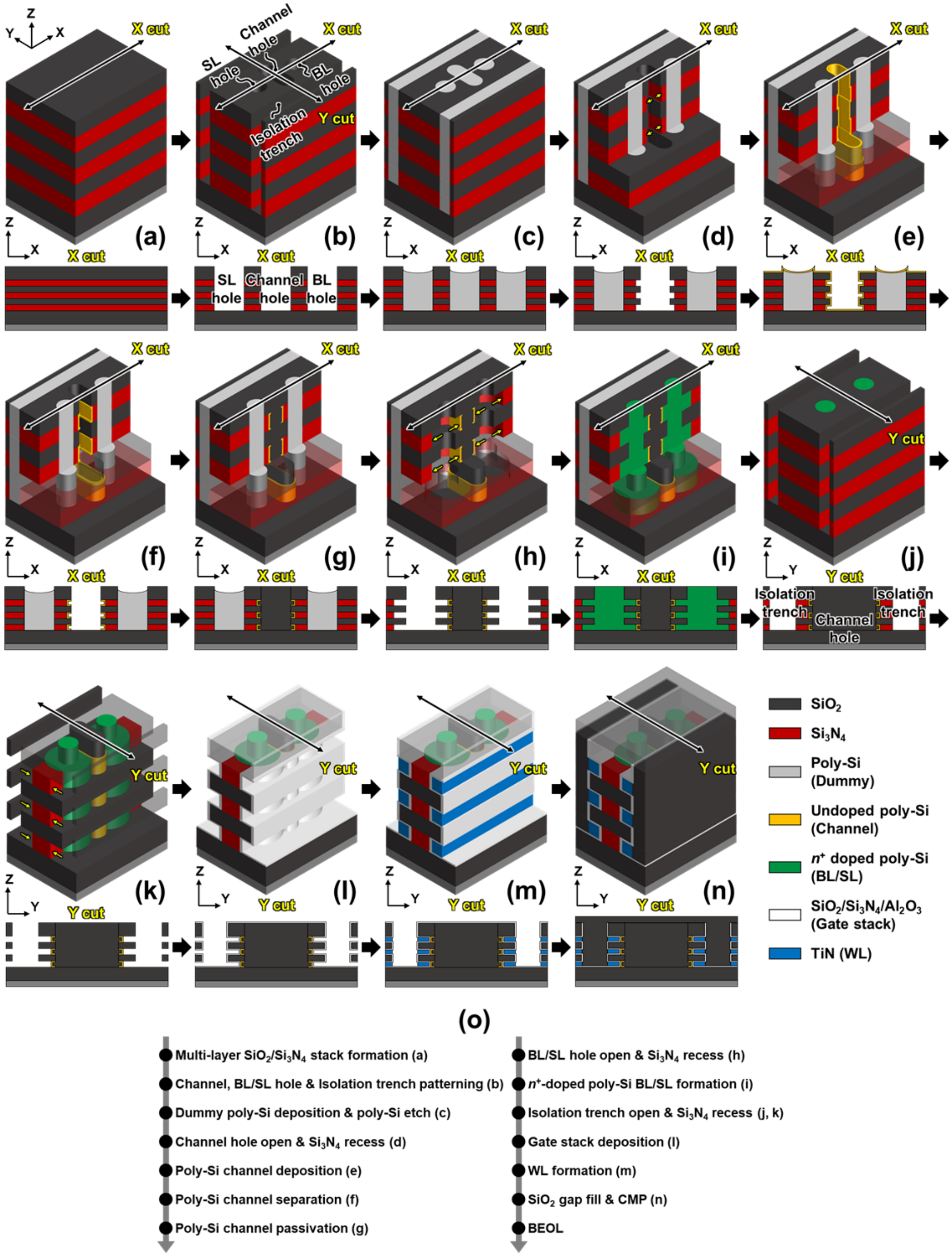

Figure S1. Schematic views of key fabrication process for proposed 3D RDC flash memory cell. (a)-(n) Schematic views of key process steps. (o) Key process steps for fabricating 3D RDC flash memory cell.

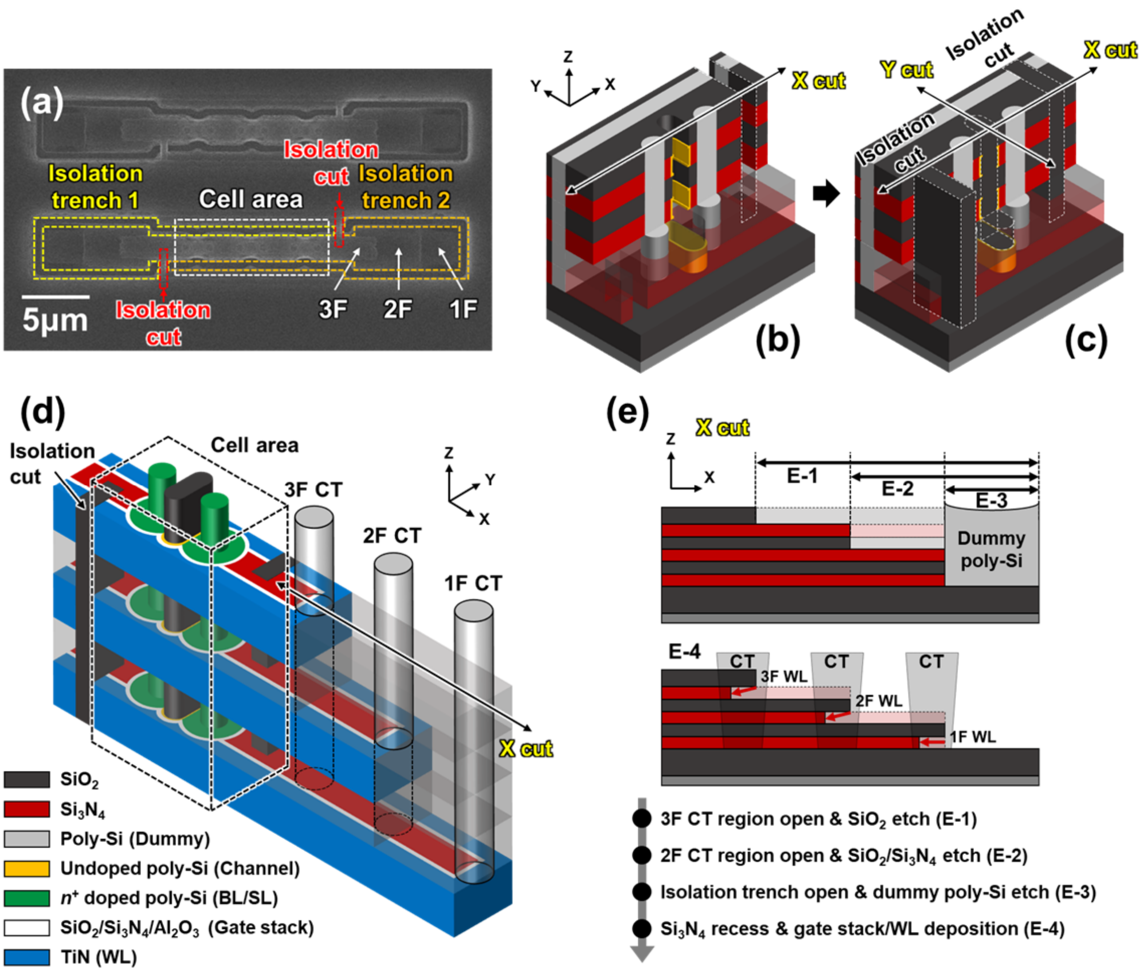

Figure S2. Key fabrication process for isolation trench separation (isolation cut) and WL contact (CT) pad formation. (a) Top SEM image of the fabricated 3D RDC flash memory cells including isolation cut patterns and WL CT pads. On both sides of the cell area, the area for the WL CT pads of each floor and isolation cut patterns exist. The isolation cut patterns separate the WL into two pieces for each floor. (b, c) Fabrication process for isolation trench separation. The channel hole and isolation cut patterns are simultaneously filled with SiO_2 and planarized. This process is performed after channel formation (Figure S1(f), Supporting Information) and before BL/SL holes open (Figure S1(h), Supporting Information). (d) Schematic view of the 3D RDC flash memory cells with three WL CT pads. Each WL CT pad provides a designated area for WL CT of each floor in the BEOL process. (e) Fabrication process for WL CT pad formation. This process is performed after BL/SL formation (Figure S1(i), Supporting Information) and before WL formation (Figure S1(m), Supporting Information).

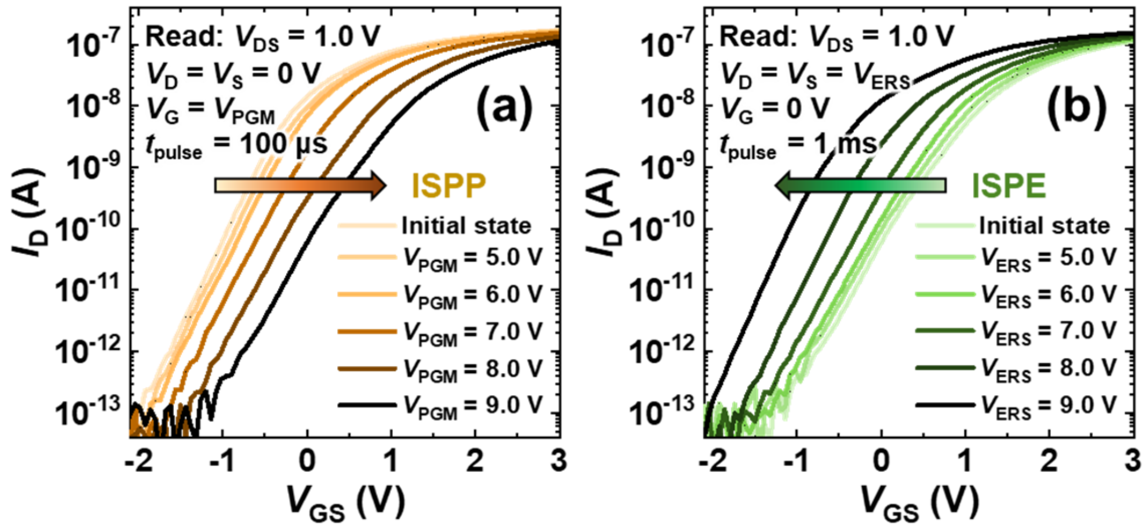

Figure S3. ISPP/ISPE characteristics of the fabricated 3D RDC flash memory cell. Transfer curves (I_D-V_{GS}) of the fabricated 3D RDC flash memory cell obtained by employing (a) ISPP and (b) ISPE schemes. The fabricated cell demonstrates low-power PGM/ERS operations.

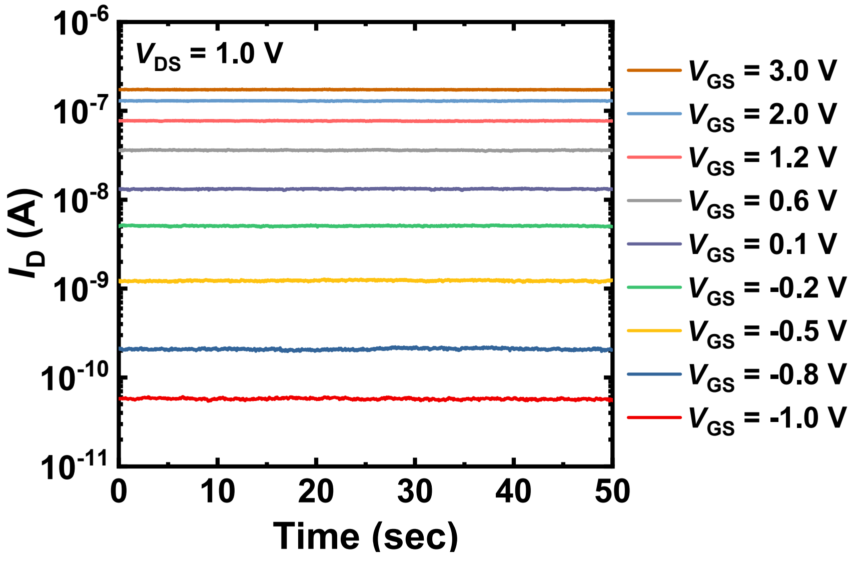

Figure S4. I_D variation of the fabricated 3D RDC flash memory cell over time. The fabricated cell exhibits stable read operation characteristics for various V_{GS} conditions.

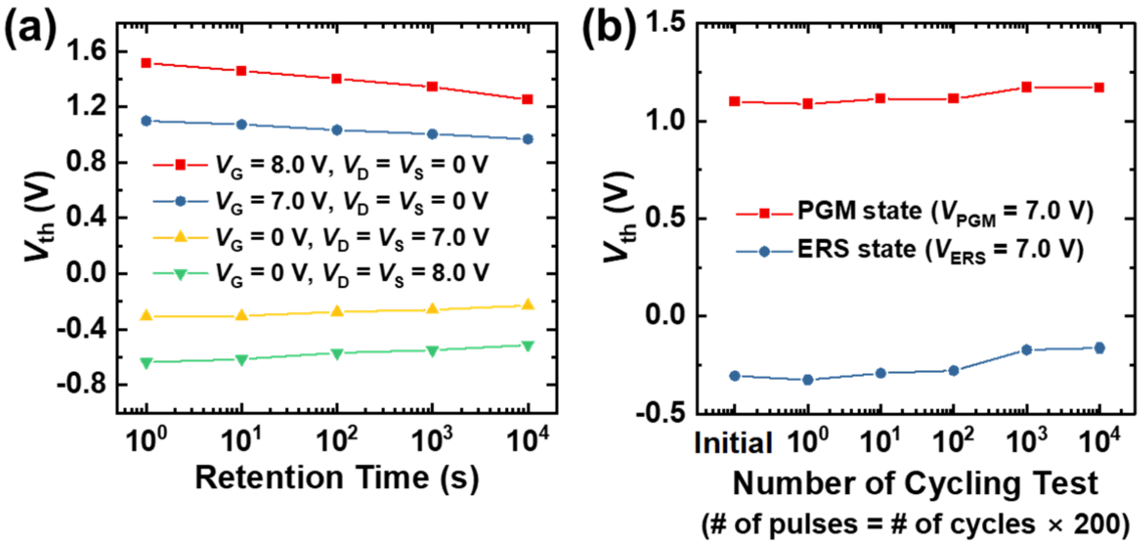

Figure S5. Retention and endurance characteristics of the fabricated 3D RDC flash memory cell. (a) Retention test of 4 conductance states. (b) Endurance test for 10^4 PGM/ERS cycles. 200 PGM/ERS pulses are applied in one cycling test. It should be highlighted that the retention characteristics can be enhanced either by removing shallowly trapped charges or by inhibiting lateral migration of charges within the charge-trapping layer [S1, S2].

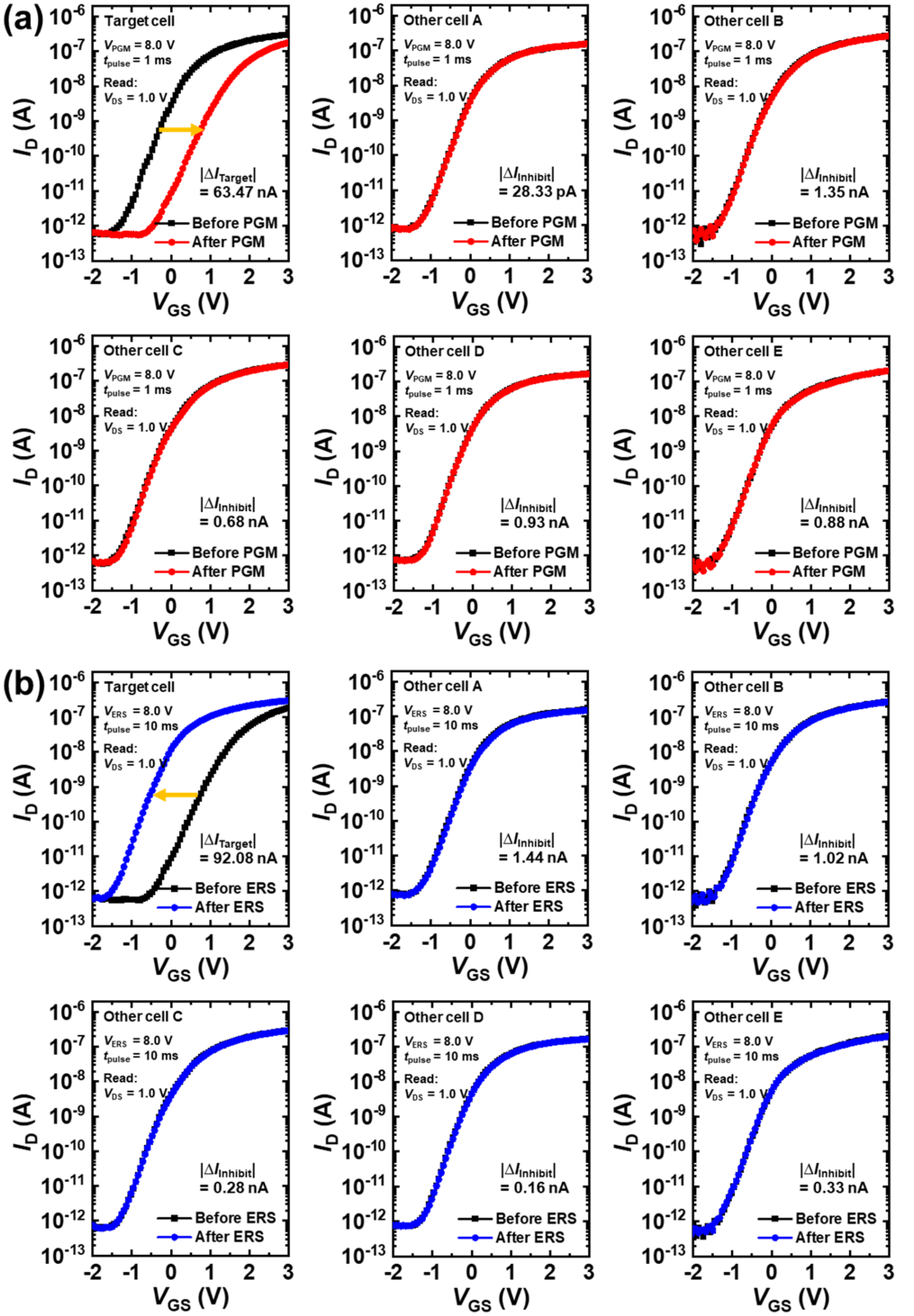

Figure S6. Random-access capability of the fabricated 3D RDC flash memory array. Transfer curves (I_D-V_{GS}) of the fabricated 3D RDC flash memory cells with the selective (a) PGM and (b) ERS operations.

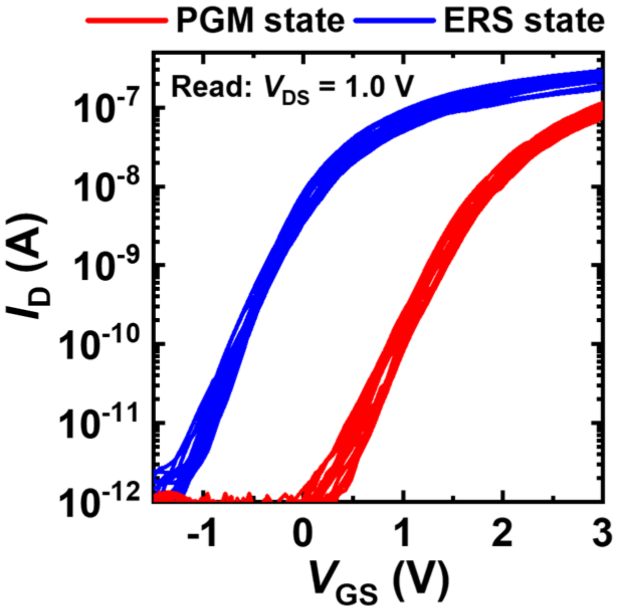

Figure S7. Transfer curves (I_D-V_{GS}) of the randomly selected 20 fabricated 3D RDC flash memory cells. Device-to-device variation exists in the fabricated cells.

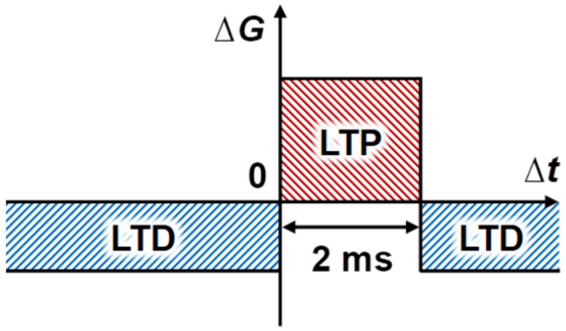

Figure S8. Simplified spike-timing-dependent plasticity (STDP) learning rule.

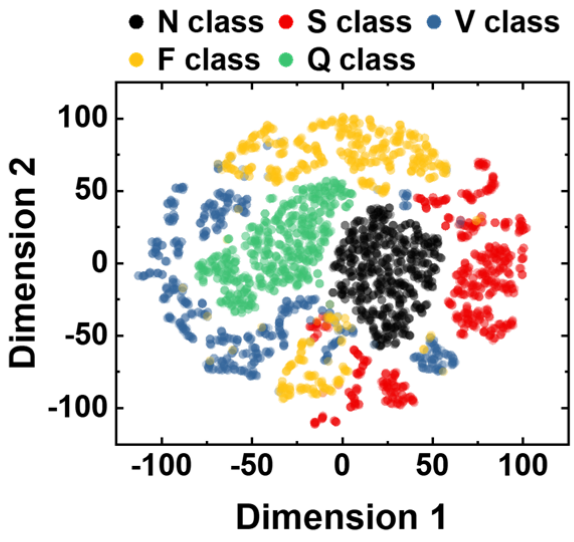

Figure S9. The t-distributed stochastic neighbor embedding (t-SNE) visualization, of the samples from the MIT-BIH dataset for the ECG classification.

Supplementary Tables

	LTP	LTD
**Model equation**	$G(n+1) = G(n) + \alpha_p exp\left(-\beta_p \frac{G(n)-G_{min}}{G_{max}-G_{min}}\right)$	$G(n+1) = G(n) - \alpha_d exp\left(-\beta_d \frac{G_{max}-G(n)}{G_{max}-G_{min}}\right)$
**Parameters**	$G_{max} = 14.78, G_{min} = 1$ (normalized)	
	$\alpha_p = 1.22$	$\alpha_d = 10.32$
	$\beta_p = 3.48$	$\beta_d = 6.39$

Table S1. Non-linearity fitting parameters for LTP/LTD characteristics of the fabricated 3D RDC flash memory cells. In the model equations, $G(n)$ denotes the conductance of the synaptic devices when n pulses are applied. G_{max} and G_{min} indicate the maximum and minimum conductance values, respectively. α_p/α_d and β_p/β_d are the fitting parameters for the LTP/LTD characteristics.

Category (AAMI EC57 standard)	Annotations
N<br>(Normal beat)	• Normal<br>• Left/right bundle branch block<br>• Atrial escape<br>• Nodal escape
S<br>(Supraventricular ectopic beat)	• Atrial premature<br>• Aberrant atrial premature<br>• Nodal premature<br>• Supraventricular premature
V<br>(Ventricular ectopic beat)	• Premature ventricular contraction<br>• Ventricular escape
F<br>(Fusion beat)	• Fusion of ventricular and normal
Q<br>(Unknown beat)	• Paced<br>• Fusion of paced and normal<br>• Unclassifiable

Table S2. Heartbeat annotations categorized into five ECG types based on the PhysioNet MIT-BIH Arrhythmia dataset and the Advancement of Medical Instrumentation (AAMI) EC57 standard.

	This work	Sci. Adv. 2020 (*13*)	Adv. Funct. Mater. 2021 (*17*)	ACS Appl. Mater. Interfaces 2023 (*18*)	npj 2D Mater. Appl. 2021 (*15*)	Adv. Electron. Mater. 2022 (*16*)	IEDM 2021 (*14*)
**Synaptic Device**	3D AND-type flash memory	1D ferroelectric organic transistor	p–n cross nanowire transistor	Pt/CeO_2/Nb–$SrTiO_3$ heterojunction memristor	MoS_2/*h*-BN heterojunction photoelectroactive memory	Solid-state electrolyte gate transistor	WO_x resistor, TiO_x memristor
**# of Conductance Steps**	100	30	20	80	100	100	9
**Network Structure**	FC	FC	4-layer CNN	8-layer CNN	FC	FC	FC
**Network Size**	24×800	187×5	Supplementary Figure in (*71*)	Supplementary Figure in (*72*)	160×5	2001×10	90×24×12×4, 4×24×2
**Training Method**	On-chip (STDP)	Off-chip (BP)	Off-chip (BP)	Off-chip (BP)	Off-chip (BP)	Off-chip (BP)	Hybrid
**Accuracy (%)**	93.5	70	94.2	93	96.1	94.8 (10 classes)	97.2 (2 classes)
**Energy Consumption (μJ)***	0.21[a], 27.39[b]	0.18[b]	123730[b]	11.34[b]	0.17[b]	200.34[b]	2.54[b]

FC = Fully connected network
BP = Backpropagation algorithm
* For inferencing a single heartbeat signal
[a] I&F neuron, [b] transimpedance amplifier (TIA)

Table S3. Benchmarking results with prior works conducting ECG classification tasks using various non-volatile memory.

Supplementary Note 1: Estimation of energy consumption of the CIM architecture

The energy consumption of the CIM architecture is quantitatively estimated as follows,

$$E_{cell} = {V_{read}}^{2} \times G_{cell} \times t_{read} = (1.0\ \mathrm{V})^{2} \times 20\ \mathrm{nS} \times 100\ \mathrm{\mu s} = 2\ \mathrm{pJ/spike} \quad , \quad (1)$$

where E_{cell} is the energy consumption of a single 3D RDC flash memory cell per read operation, V_{read} is the read voltage, G_{cell} is the conductance of the flash memory cell, and t_{read} is the width of the read pulse. We assume an average G_{cell} of 20 nS for the fabricated flash memory cells. The proposed CIM architecture with 24 input neurons and 800 output neurons requires $24 \times 800 = 19200$ synaptic weights. The energy consumption to drive the 3D RDC flash memory array (E_{array}) can be calculated as follows,

$$E_{array} = n_{cell} \times E_{cell} = 19200 \times 2\ \mathrm{pJ/spike} = 38.4\ \mathrm{nJ/spike} \quad , \quad (2)$$

where n_{cell} is the required number of synaptic weights. Meanwhile, the energy consumption of the I&F neuron circuit ($E_{I\&F}$) is estimated by [S3] as follows,

$$E_{I\&F} = n_{neuron} \times 5.3\ \mathrm{pJ/spike} = 800 \times 5.3\ \mathrm{pJ/spike} = 4.24\ \mathrm{nJ/spike} \quad , \quad (3)$$

where n_{neuron} is the number of neuron circuits. By adding E_{array} and $E_{I\&F}$, we calculate the total energy consumption of the 3D RDC flash memory array (E_{total}) for inferencing a single heartbeat signal as follows,

$$E_{total} = n_{spike} \times \left(E_{array} + E_{I\&F}\right) = 5 \times 42.64\ \mathrm{nJ/spike} = 0.21\ \mathrm{\mu J} \quad , \quad (4)$$

when assuming the average number of spikes (n_{spike}) is 5 out of a maximum number of 10. The proposed CIM architecture requires an energy consumption of 0.21 μJ for inferencing a single heartbeat signal.

In other works using a backpropagation (BP) algorithm, two synaptic devices per synaptic weight are required to express negative synaptic weights. The transimpedance amplifiers (TIA), low-power analog circuits, are used for energy consumption estimation of the other works [S4, S5]. The same values are used for all other variables.

References

[S1] S.-H. Park, D. Kwon, H.-N. Yoo, J.-W. Back, J. Hwang, Y. Yang, J.-J. Kim, J.-H. Lee, "Retention Improvement in Vertical NAND Flash Memory Using 1-bit Soft Erase Scheme and its Effects on Neural Networks" in *IEEE International Electron Devices Meeting, IEDM* (2022).

[S2] H.-J. Kang, N. Choi, D. H. Lee, T. Lee, S. Chung, J.-H. Bae, B.-G. Park, J.-H. Lee, "Space program scheme for 3-D NAND flash memory specialized for the TLC design" in *IEEE Symposium on VLSI Technology* (2018).

[S3] K.-H. Lee, D. Kwon, S. Y. Woo, J. H. Ko, W. Y. Choi, B.-G. Park, J.-H. Lee, Highly Linear Analog Spike Processing Block Integrated With an AND-Type Flash Array and CMOS Neuron Circuits. *IEEE Transactions on Electron Devices*. **69**, 6065–6071 (2022).

[S4] A. Atef, M. Atef, M. Abbas, E. E. M. Khaled, "High-sensitivity regulated inverter cascode transimpedance amplifier for near infrared spectroscopy" in *2016 Fourth International Japan-Egypt Conference on Electronics, Communications and Computers (JEC-ECC)*. 99–102 (2016).

[S5] P. Lin, C. Li, Z. Wang, Y. Li, H. Jiang, W. Song, M. Rao, Y. Zhuo, N. K. Upadhyay, M. Barnell, Q. Wu, J. J. Yang, Q. Xia, Three-dimensional memristor circuits as complex neural networks. *Nature Electronics*. **3**, 225–232 (2020).
